# Functionalization of kaolinite for removal of phosphate from urban sewage

**DOI:** 10.1016/j.mex.2021.101423

**Published:** 2021-06-18

**Authors:** Camilla Carbinatti, Fabiano Tomazini da Conceição, Rodrigo Braga Moruzzi, Amauri Antônio Menegário

**Affiliations:** a UNESP - Universidade Estadual Paulista, Instituto de Geociências e Ciências Exatas, Avenida 24-A, no 1515, CEP 13506-900, Bela Vista, Rio Claro, São Paulo, Brazil;; b UNESP - Universidade Estadual Paulista, Centro de Estudos Ambientais, Rio Claro, Brazil.

**Keywords:** Clay minerals, Surface functionalization, Phosphate removal, Mineral precipitation

## Abstract

The PO_4_^3−^ widespread in urban sewages promotes eutrophication of water sources, with harmful effects to natural life and endanger human health. The removal of PO_4_^3−^ from urban sewage requires treatment at tertiary level, with high costs and low efficiency in most cases. Thus, a functionalization method for surface modification of kaolinite was proposed to improve the removal of PO_4_^3−^ from urban sewage. The kaolinite commercial did not remove PO_4_^3-^ from aqueous solution. However, the functionalized kaolinite (FK) was efficient, with a maximum removal capacity of 8.4 ± 0.1 mg PO_4_^3−^/L, within less than 1 min of reaction. The removal of PO_4_^3-^ is associated with precipitation of pyromorphite, a mineral with low solubility (*K_sp_* < 10^−79,6^). Finally, real urban sewage samples (raw and treated) were also tested for removal of PO_4_^3-^ using FK, confirming its effectiveness. The central aspects of this development are:

• Functionalized kaolinite (FK), with Pb(II), for removal of PO_4_^3−^ from urban sewage was studied.
• The FK was efficient for removal of up to 8.4 mg PO_4_^3−^/L from aqueous solution, within a short reaction time.
• The precipitation of pyromorphite was the mechanism responsible for removal of PO_4_^3-^ and FK efficiency have been confirmed for real urban sewage samples.

## Graphical Abstract

**Figure:**
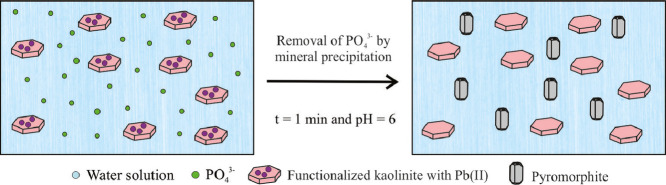

Specifications table

**Table:** 

Subject Area:	Environmental Science
More specific subject area:	Removal of phosphate from urban sewage
Method name:	Functionalization of kaolinite for removal of phosphate from urban sewage
Name and reference or original method:	S. Moharami & M. Jalali (2015). Use of modified clays for removal of phosphorous from aqueous solutions. Environ. Monit. Assess. 187:639.
Resources availability:	N/A

## Method details

### Background

The PO_4_^3−^ present in urban sewage promotes eutrophication in the water bodies [1]. This anion is difficult to remove during the treatment of urban sewage, requiring treatment at the tertiary level [2]. The most used method for the removal of PO_4_^3−^ from urban sewage is the chemical precipitation, involving the addition of bivalent or trivalent metal salts [3], [4], [5]. Recently, studies have shown the precipitation of pyromorphite (5Pb^2+^ + 3PO_4_^3−^ + H_2_O → Pb_5_(PO_4_)_3_(OH)_(pyromorphite)_ + H^+^) in natural surface waters due to presence of PO_4_^3−^ and Pb(II), reducing the concentration of dissolved Pb(II) [6,7]. The pyromorphite has a low solubility constant (*K_sp_* < 10^−79,6^), preventing that the Pb(II) returns to the environment as a dissolved cation [8], [9], [10].

Kaolinite [Al_2_Si_2_O_5_(OH)_4_] has its negatively-charged surface, becoming this mineral an important adsorbent for cationic ions [11]. The functionalization of kaolinite with acid treatment [12] and bivalent trace elements can promote the removal of anionic molecules, such as PO_4_^3−^, present in the urban sewage [13], [14], [15]. Based on the functionalization of commercial kaolinite (CK) with bivalent trace elements, the functionalized kaolinite (FK) with Pb(II) was produced. The efficiency for removal of PO_4_^3-^ from aqueous solution using FK was studied and compared with CK. Furthermore, the reaction time and maximum removal capacity of PO_4_^3-^ was determined using FK. Finally, the FK was applied in real urban sewage samples, attesting its effectiveness.

### Functionalization of commercial kaolinite

The CK (Sigma-Aldrich®, CAS Number 1318-74-7) was used in this study. The following procedures have been applied for the functionalization:

1 – About 1.0 g of CK was placed in a beaker;
2 – 10 mL of aqueous solution with Pb(II) initial concentration of 40 mg/L was added;
3 – The beaker was agitated (digital shaker Biothec – model BT 645) for 24 h at 145 rpm;
4 – The solution was centrifuged (centrifuge Excelsa II® – model 206-BL) for 25 min at 3000 rpm;
5 – The FK was separated and washed three times, using ultrapure water (Milli-Q® system - model IQ 7000) with electrical conductivity lower than 0.02 µS/cm;
6 – Finally, the FK was dried for 12 h at 40 °C.

## Method validation

For validation purposes, 1.0 g of each sample (CK and FK) was mixed with 10 mL of aqueous solution containing PO_4_^3−^ in the initial concentration (*C_0_*) of 1 mg/L at pH 6 [9]. The suspension was shaken for 24 h at 145 rpm, and then centrifuged at 3000 rpm for 25 min. The supernatant was separated and the PO_4_^3-^ concentration remaining in solution (*C_e_*) was measured using a Hach DR-2800 spectrophotometer, with a detection limit of 0.1 mg/L. The removal efficiency of PO_4_^3-^ (*%A* – in percentage) was determined according to the Eq. 1. The experimental procedures were carried out in triplicate.

(1) $$\%A=\left[\left(C_{0}--C_{e}\right)/C_{e}\right].100$$

**Fig. 1:**
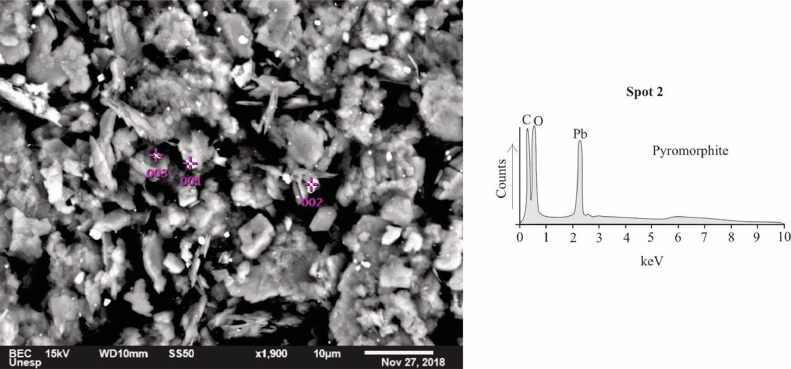
SEM-EDS images of functionalized kaolinite after the removal of PO_4_^3−^, showing the precipitation of small grains (lower than 5 µm) of pyromorphite (spots 1, 2 and 3).

The results are presented in Table 1. The removal efficiencies of PO_4_^3−^ were 0 and 100% using CK and FK, respectively. These results evidenced the functionalization plays a crucial role on the removal of PO_4_^3−^. The precipitation of pyromorphite was the main mechanism associated with PO_4_^3-^ removal using FK with Pb(II), as shown in Fig. 1.

**Table 1:** Removal efficiency (*%A*) of PO_4_^3−^ (mg/L) using FK and CK in aqueous solution.

CK			FK		
*C_0_*	*C_e_*	*%A*	*C_0_*	*C_e_*	*%A*
1.0	1.0 ± 0.1	0	1.0	< 0.1	100

## Reaction time and maximum removal capacity of PO_4_^3-^ using functionalized kaolinite

The reaction time for removal of PO_4_^3-^ using KF has been investigated, according the following procedures carried out in triplicate. The FK (1.0 g) was mixed in 10 mL of an aqueous solution with *C_0_* of 1 mg PO_4_^3−^/L at pH 6 [9]. The suspension was shaken at 145 rpm, with samples taken after 1, 5, 15, 30 and 60 min. The solution was centrifuged at 3000 rpm for 25 min, with the supernatant separated and the *C_e_* determined. The experiments have shown no residuals of PO_4_^3-^ after 1 min of reaction time (Table 2), showing a fast reaction time for removal of PO_4_^3−^ associated to the mineral pyromorphite precipitation.

**Table 2:** Reaction time (min) and removal efficiency (*%A*) of PO_4_^3−^ (mg/L) using FK in aqueous solution.

Time	C_0_	C_e_	%A
0	1.0	1.0 ± 0.1	0
1	1.0	< 0.1	100
5	1.0	< 0.1	100
15	1.0	< 0.1	100
30	1.0	< 0.1	100
60	1.0	< 0.1	100

The maximum removal capacity of PO_4_^3−^ using FK was also determined (in triplicate). The samples with 1.0 g: 10 mL of an aqueous solution with *C_0_* of 1 mg/L were stirred at 145 rpm for 5 min at pH 6 [9], with *C_0_* of 1, 2, 3, 4, 7 and 9 mg/L. The solutions were centrifuged for 25 min at 3000 rpm and the *C_e_* determined in the supernatants. The maximum removal capacity of PO_4_^3−^ using FK was 8.4 ± 0.1 mg/L (Table 3) or 8.4 mg/g, indicating an efficiency of 93.3% for removal of PO_4_^3-^ from aqueous solutions with *C_0_* of 9 mg/L. The value of 8.4 mg/g is higher than the removal capacities obtained for natural or functionalized kaolinite, i.e., CK used in this study (< 0.1 mg/g), kaolinite from Linthipe (ca. 0.15 mg/g) [12], modified kaolinite with FeCl_3_ (1.31 mg/g) [13] and modified kaolinite with seawater in different temperatures (4.07 mg/g at 600 °C) [15].

**Table 3:** Maximum removal capacity of PO_4_^3−^ using FK and concentration of residual Pb(II) in aqueous solution.

PO_4_^3-^ (mg/L)			Pb(II) (mg/L)
*C_o_*	*C_e_*	*%A*	
1	< 0.1	100	692 ± 20
2	< 0.1	100	91 ± 4
3	< 0.1	100	63 ± 3
4	< 0.1	100	< 0.006
7	< 0.1	100	< 0.006
9	0.6 ± 0.1	93.3	< 0.006

Trace levels of residual Pb(II) in the treated effluent can pose a serious environmental risk for aquatic systems due to its toxicity. Thus, the concentration of Pb(II) were also determined in the supernatants by inductively coupled plasma optical emission spectrometry (ICP OES), iCAP 6000 SERIES machine Thermo Scientific, with detection limit of 0.006 mg/L. The presence of residual Pb(II) in aqueous solutions was measured in aqueous solutions with *C_0_* of 1, 2 e 3 mg/L of PO_4_^3−^. Residual Pb(II) was not detected for *C_0_* of PO_4_^3−^ ≧ 4 mg/L, indicating the use of FK for removal of PO_4_^3−^ only in urban sewage with *C_0_* ≧ 4 mg/L. Further studies for lower *C_0_* concentrations in urban sewage are encouraged before application.

## Removal of PO_4_^3-^ using functionalized kaolinite in real urban sewage samples

Three samples of raw and treated urban sewage were collected in a wastewater treatment plant (WWTP) located in Rio Claro, São Paulo State, Brazil. These samples were stored in labeled amber container at 4°C and transported immediately to the laboratory, where they were filtered, using 0.45 μm MF-Millipore® membrane filter, and the *C_0_* of PO_4_^3−^ and Pb(II) measured (Table 4). In order to verify the real removal efficiency of PO_4_^3−^ from urban sewage (raw and treated), 10 mL of each filtered sample were mixed with 1.0 g of FK. The solutions were shaken at 145 rpm for 5 min at pH 6 [9], centrifuged at 3000 rpm for 25 min, and then the *C_e_* of PO_4_^3−^ and Pb(II) were determined in the supernatants (Table 4).

**Table 4:** *C_0_* and *C_e_* averages of PO_4_^3-^ and Pb(II) (in mg/L) measured in the urban sewage collected in a WWTP located in Rio Claro.

Sample	*C_0_* of PO_4_^3−^	*C_e_* of PO_4_^3−^	*C_0_* of Pb(II)	*C_e_* of Pb(II)
Raw	6.1 ± 0.1	< 0.1	< 0.006	< 0.006
Treated	3.8 ± 0.1	< 0.1	< 0.006	< 0.006

The *C_0_* averages of PO_4_^3-^ were 6.1 ± 0.1 e 3.8 ± 0.1 mg/L, respectively, for raw and treated urban sewage. After the use of FK, *C_e_* averages of PO_4_^3-^ were lower than 0.1 mg/L. In addition, the *C_0_* and *C_e_* averages of Pb(II) were always lower than the detection limit of 0.006 mg/L. These results show the efficiency during the use of FK for removal of PO_4_^3−^ from urban sewage in real samples collected in a WWTP.

## Conclusions

A method for functionalization of kaolinite for removal of PO_4_^3−^ from urban sewage was studied. The functionalized kaolinite (FK) with Pb(II) have shown a promising alternative for removal of PO_4_^3−^ in aqueous solution, with maximum removal capacity of 8.4 mg/L, within a reaction time lower than 1 min. The precipitation of PO_4_^3-^ is associated with pyromorphite, a mineral with low solubility (*K_sp_* < 10^−79,6^). Finally, real urban sewage samples (raw and treated) were also tested for removal of PO_4_^3-^ using KF, confirming its effectiveness for removal of PO_4_^3−^ from urban sewage with *C_0_* ≧ 4 mg/L.
